# Gaze palsy, hypogeusia and a probable association with miscarriage of pregnancy - the expanding clinical spectrum of non-opticospinal neuromyelitis optica spectrum disorders: a case report

**DOI:** 10.1186/s13104-015-0991-5

**Published:** 2015-02-10

**Authors:** Thashi Chang, Milinda Withana

**Affiliations:** Department of Clinical Medicine, Faculty of Medicine, University of Colombo, 25, Kynsey Road, Colombo, 08 Sri Lanka; University Medical Unit, National Hospital of Sri Lanka, Colombo, Sri Lanka

**Keywords:** NMOSD, Gaze palsy, Hypogeusia, Area postrema, Miscarriage

## Abstract

**Background:**

Neuromyelitis optica is characterised by optic neuritis, longitudinally-extensive transverse myelitis and presence of anti-aquaporin-4 antibodies in the serum. However, non-opticospinal central nervous system manifestations have been increasingly recognised. Awareness of the widening clinical spectrum of neuromyelitis optica (unified within the nosology of ‘neuromyelitis optica spectrum disorders’) is key to earlier diagnosis and appropriate therapy. We report 2 patients to illustrate the varied clinical manifestations of neuromyelitis optica spectrum disorders while postulating an effect of anti-aquaporin-4 antibodies on the miscarriage of pregnancy. This is the first report of horizontal gaze palsy as a presenting symptom of neuromyelitis optica spectrum disorders.

**Case presentation:**

Patient 1

A 17-year-old Sri Lankan female presented with hypersomnolence, lateral gaze palsy and loss of taste of 1 week duration. Two years previously she had presented with intractable hiccups and vomiting followed by a brainstem syndrome. Magnetic resonance imaging showed a lesion in the left cerebellum extending into the pons while lesions in bilateral hypothalami and medulla noted 2 years ago had resolved. Autoimmune, vasculitis and infection screens were negative. Anti-aquaporin-4 antibodies were detected in serum. All her symptoms resolved with immunosuppressive therapy.

Patient 2

A 47-Year-old Sri Lankan female presented with persistent vomiting lasting over 3 weeks. Three years previously, at 25-weeks of her 4^th^ pregnancy, she had presented with quadriparesis and was found to have a longitudinally extensive transverse myelitis from C2 to T2 vertebral levels, which gradually improved following intravenous steroid therapy. Magnetic resonance imaging showed a hyper-intense lesion in the area postrema and longitudinally extensive atrophy of the cord corresponding to her previous myelitis. Autoimmune, vasculitis and infection screens were negative. Anti-aquaporin-4 antibodies were detected in serum. Her vomiting subsided with immunosuppressive therapy. Her second pregnancy had resulted in a first-trimester miscarriage.

**Conclusion:**

The clinical spectrum of neuromyelitis optica spectrum disorders has expanded beyond optic neuritis and myelitis to include non-opticospinal syndromes involving the diencephalon, brainstem and cerebrum. Our report highlights the varied central nervous system manifestations of neuromyelitis optica spectrum disorders and miscarriage of pregnancy possibly related to anti-aquaporin-4 antibodies.

**Electronic supplementary material:**

The online version of this article (doi:10.1186/s13104-015-0991-5) contains supplementary material, which is available to authorized users.

## Background

Neuromyelitis optica (NMO) was first described by Devic and Gault in 1894 as a monophasic disorder characterised by bilateral (or rapidly sequential) optic neuritis and myelitis [1,2]. Its relapsing nature was noted in the 20^th^ century [3] while the discovery of highly-specific anti-aquaporin-4 (AQP4) antibodies established NMO as a distinct disease [4], which required the presence of optic neuritis and myelitis for diagnosis [5]. However, since then more restrictive and more extensive central nervous system (CNS) involvement in NMO has been recognised and revised diagnostic criteria have been proposed [6]. The new criteria define a unifying diagnosis of ‘NMO spectrum disorders’ (NMOSD) which requires only one core clinical characteristic in patients seropositive for AQP4 antibodies. The core clinical characteristics include optic neuritis, acute myelitis, area postrema syndrome, acute brainstem syndrome, diencephalic syndrome and symptomatic cerebral syndrome with typical brain lesions. The new criteria have widened the clinical spectrum that encompasses a diagnosis of NMOSD and thus demands a high index of clinical suspicion in patients who present with non-opticospinal CNS manifestations. We report 2 patients to illustrate the varied clinical manifestations of NMOSD while presenting the first report of horizontal gaze palsy as a presenting symptom of NMOSD.

## Case presentation

### Patient 1

A 17-year-old Sri Lankan female presented with daytime hypersomnolence, lassitude, featureless persistent dull headache and loss of taste of 1 week duration.

**Figure 1:**
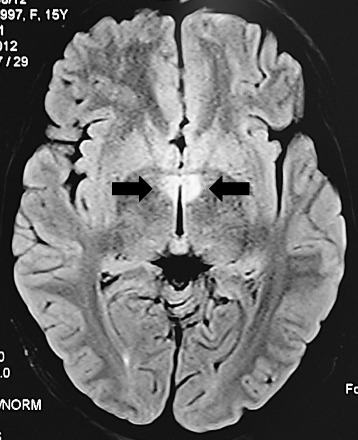
**T2-weighted fluid attenuated inversion recovery (FLAIR) MRI showing high signal intensity lesions in both hypothalami (arrows).**

Two years previously, she had presented for the first time with intractable hiccups and vomiting that lasted a week following a febrile episode and required insertion of a nasogastric tube. Magnetic resonance imaging (MRI) of the brain had shown high signal intensities on T2 and FLAIR sequences in the region of hypothalami bilaterally with no restricted diffusion (Figure 1). CSF analysis had shown normal protein and glucose but with 15 cells/cumm of which 10 were polymorphs. Screening for infections and vasculitis including anti-nuclear antibodies (ANA), anti-neutrophil cytoplasmic antibodies, erythrocyte sedimentation rate (ESR), c-reactive protein (CRP) were normal. She was thought to have a post-viral syndrome and treated supportively and discharged from hospital after 6 days.

**Figure 2:**
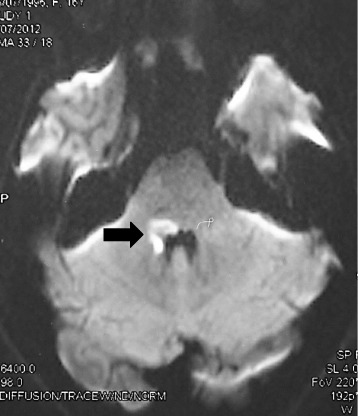
**Diffusion-weighted magnetic resonance imaging showing restricted diffusion in the right middle cerebellar peduncle (arrow).** The lesion extended from the pons to the posterior spinal cord (not shown).

One month later, she had presented again with ataxia and was found to have a right lateral rectus palsy and nystagmus. Repeat MR imaging of the brain showed no interval change from the previous scan whilst further screening for a paraneoplastic aetiology with computerised tomography (CT) of chest, abdomen and pelvis proved negative. Repeated autoimmune screening was negative. She was suspected to have a Wernicke syndrome and treated with intravenous thiamine. She left hospital a week later. A follow up MRI brain done one month later, had shown abnormal areas of hyperintensity signals on FLAIR and T2-weighted sequences and slight hypointensity on T1 weighted sequences in the medulla oblongata extending to the proximal spinal cord, right side of the pons, the middle cerebellar peduncle and anterior inferior thalami extending to the hypothalami (Figure 2).

Her symptoms had gradually improved and she had returned to her normal daily activities including schooling until her current presentation. Apart for being diagnosed with hypothyroidism and being medicated with 75 micrograms of thyroxine daily in the last 2 years, her past medical history was unremarkable.

**Figure 3:**
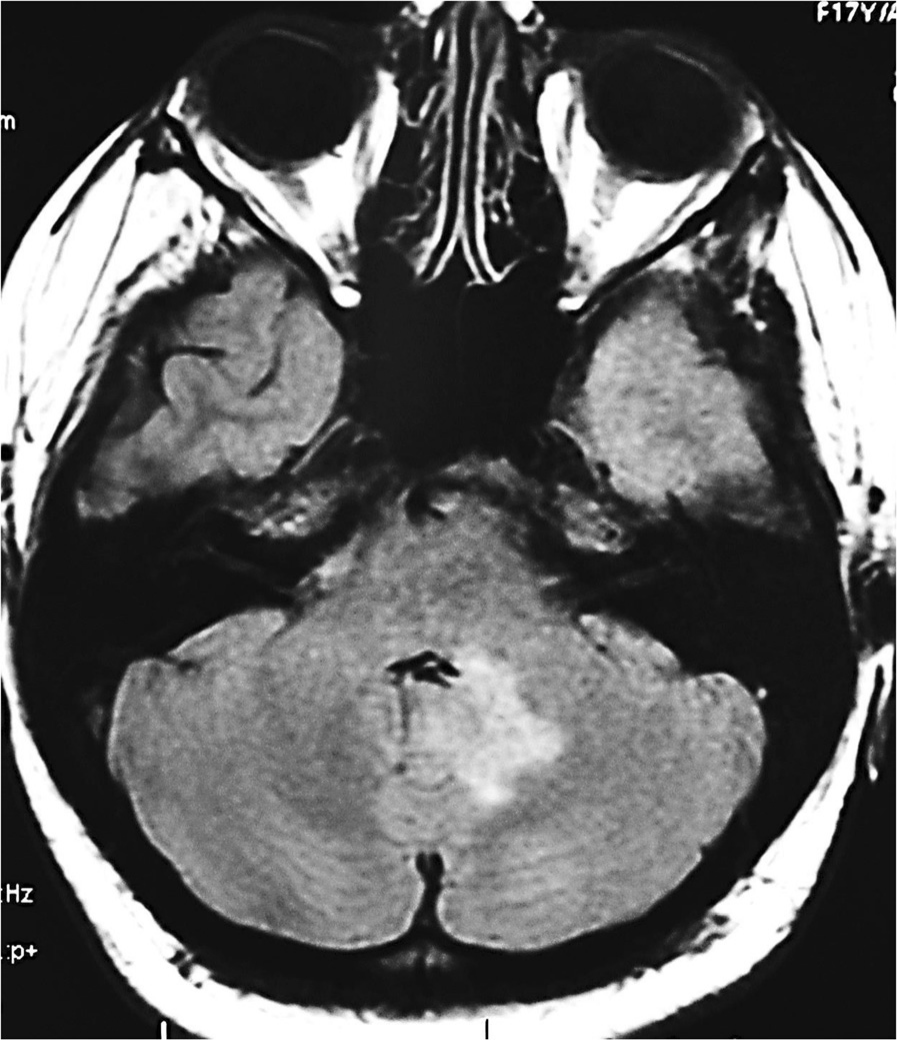
**T2-weighted FLAIR magnetic resonance imaging showing high signal intensity lesion in the left cerebellar hemisphere extending into the middle cerebellar peduncle and left dorsal pons.**

On examination, she was found to have a gaze palsy to the left and nystagmus more prominent when looking to the right. Taste for all modalities was significantly impaired more on the left than right side of her tongue. The rest of the examination was normal. MRI brain showed a new T2-hyperintense lesion in the left cerebellar hemisphere extending to the left middle cerebellar peduncle and dorsal pons with no gadolinium enhancement (Figure 3). Previously noted lesions had completely resolved. Repeated ANA was negative and thyroid stimulating hormone was within normal limits. Visual evoked potentials were normal bilaterally. Serum NMO IgG was positive (cell-based assay).

She was treated with 1g of intravenous methyl prednisolone daily for 5 days and discharged on oral prednisolone at 1 mg/kg/d. At one week review, daytime hypersomnolence had resolved, her taste was reported to be slightly better and her eye movements noted to have improved, but with persistent gaze palsy and nystagmus. At one month review, her eye movements were full and normal, and taste sense completely normal. Mycophenolate mofetil was introduced whilst a slow taper of prednisolone was commenced.

### Patient 2

A 47-Year-old Sri Lankan female presented with persistent vomiting lasting more than three weeks with 5 – 6 episodes per day. Vomitus consisted of food particles and occasionally was bile stained. Vomiting was not associated with fever, colicky abdominal pain, distention, nausea or regurgitation. Bowel habits remained usual. She did not report any other neurological symptom. Clinical examination was unremarkable except for quadrihyperreflexia and bilateral extensor plantar responses. Haematological and biochemical screening including blood counts, renal and liver function tests were normal. Inflammatory markers (ESR and CRP) were normal while ANA was negative. Ultrasonography of abdomen and endoscopy of upper gastrointestinal tract were normal. Serum NMO IgG was positive (cell-based assay).

Three years previously, at 25-weeks of her fourth pregnancy, she had presented with quadriparesis (power 4/5) and was found to have a longitudinally extensive transverse myelitis from C2 to T2 vertebral levels for which extensive investigations did not reveal an infectious, neoplastic or autoimmune aetiology. NMO IgG had not been tested at that time. She gradually regained normal limb power over a period of 12 weeks subsequent to being treated with intravenous methyl prednisolone pulses followed by a tapering regimen of oral prednisolone. Her second pregnancy had resulted in a first-trimester miscarriage for which no cause had been found including a negative screen for systemic lupus erythematosus and anti-phospholipid antibody syndrome

**Figure 4:**
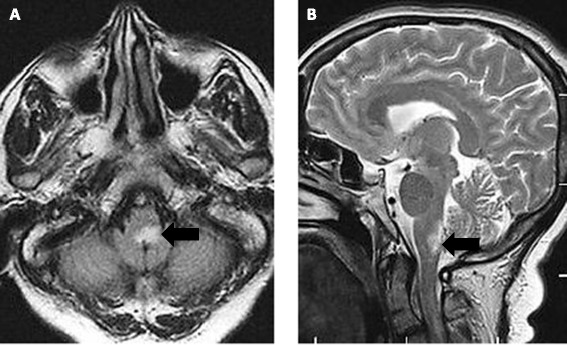
**Axial (A) and sagittal (B) T2-weighted FLAIR magnetic resonance imaging showing high signal intensity lesion in the dorsal medulla corresponding to the area postrema (arrows).**

MRI showed a hyper-intense lesion in the dorsal medulla (Figure 4) and longitudinally extensive atrophy of the cord corresponding to her previous myelitis.

Her vomiting subsided following treatment with intravenous methyl prednisolone. She was discharged on oral prednisolone 1 mg/kg and mycophenolate mofetil 500 mg BD. At 2-weeks review she remained asymptomatic.

## Conclusions

We describe two patients with seropositive NMOSD: one with a diencephalic-brainstem syndrome and the other with a myelitis-area postrema syndrome illustrating the widening clinical presentations of non-opticospinal-NMOSD. Both patients demonstrated relapsing disease with dissemination in space. Patient 1 had recurrent non-opticospinal lesions while patient 2 developed sequentially two of the commonest core clinical characteristics of NMOSD [6].

Diencephalic presentations characterised by hypersomnolence and hypothalamic lesions have been previously reported [7,8]. However, only two previous reports of hypogeusia as a feature of NMOSD exists in the literature [9,10] while horizontal gaze palsy has not been previously reported in NMOSD. Patient 1 presented with horizontal gaze palsy, hypogeusia and hypersomnolence, all of which completely resolved with immunosuppressive treatment. The MRI lesion extending into the midpontine tegmentum suggests interruption of the gustatory pathway to account for her hypogeusia [9] while further extension of the lesion into the pons would involve the parapontine reticular formation to account for her lateral gaze palsy although it was not evident in the current study. Furthermore, although she presented with hypersomnolence, no lesions were noted in the diencephalon. It is conceivable, that the early timing of the brain imaging may have missed these evolving lesions. This postulation is supported by the observation that medullary lesions were detected only in subsequent MRIs when she initially presented with an area postrema syndrome two years ago. Interestingly, in the natural course of her disease new lesions occurred after 1 month and 2 years following the initial presentation and the initial lesions resolved without immunosuppressive treatment.

Patient 2 presented with an area postrema syndrome and a corresponding lesion in the dorsal medulla. Area postrema syndrome is now recognised as one of the commonest core clinical characteristics of NMOSD in addition to optic neuritis and longitudinally extensive transverse myelitis (LETM) and has been included in the proposed new diagnostic criteria [6]. Lack of awareness of its occurrence in NMOSD is likely to provoke a search for a gastrointestinal aetiology for persistent vomiting. Patient 2 had a LETM three years previously, but none of the core clinical characteristics described in NMOSD are pathognomonic and LETM may occur in infections, neoplasia and autoimmune/inflammatory diseases of the CNS [11]. However, relapses with core clinical lesions in the CNS with dissemination in time are sufficient for a diagnosis of NMOSD even in the absence of AQP4 antibodies [6]. Her obstetric history is noteworthy since there is evidence to suggest that NMO-IgG can cause miscarriage through complement-induced placentitis [12]. It is tempting to postulate that she had AQP4 antibodies at a level that was sufficient to cause abortion in her 2^nd^ pregnancy and a relapsed rise of the antibody titre sufficient to cause a CNS syndrome in her 4^th^ pregnancy given the known tendency of AQP4 antibodies to fluctuate and manifest during pregnancy and to precede neurological disease onset by as long as 16 years [13]. However, since her AQP4 antibodies had not been tested previously this association would only remain a possibility in patient 2 inciting the curiosity that AQP4 antibodies are capable of causing extra-CNS disease.

In retrospect, it could be argued that both patients should have been diagnosed in the initial presentation and long-term immunosuppressive therapy instituted to prevent relapses. However, the clinical spectrum of NMOSD has been evolving over the years since its recognition as a distinct disease in 2004 [4] and the previous diagnostic criteria lacked sensitivity in diagnosing non-opticospinal NMO syndromes. The proposed new diagnostic criteria [6] are likely to overcome the deficits of the previous criteria and facilitate earlier and more accurate diagnosis with more patients being tested for AQP4 antibodies at initial presentation.

The clinical spectrum of NMOSD has expanded beyond optic neuritis and myelitis to include non-opticospinal syndromes involving the diencephalon, brainstem and cerebrum. Our report highlights the varied CNS manifestations of NMOSD and endorses the need for the new diagnostic criteria which have expanded to incorporate CNS syndromes beyond the optico-spinal syndrome.

## Consent

Written informed consent was obtained from both patients and the legal guardian(s) of patient 1, for publication of this Case Report and the accompanying images. A copy of the written consent is available for review by the Editor-in-Chief of this journal.

## Abbreviations

AQP4: Aquaporin-4
ANA: Anti-nuclear antibodies
ANCA: Anti-neutrophil cytoplasmic antibodies
CT: Computerised tomography
CNS: Central nervous system
CRP: C-reactive protein
ESR: Erythrocyte sedimentation rate
FLAIR: Fluid attenuated inversion recovery
IgG: Immunoglobulin G
LETM: Longitudinally extensive transverse myelitis
MRI: Magnetic resonance imaging
NMO: Neuromyelitis optica
NMOSD: Neuromyelitis optica spectrum disorders
ON: Optic neuritis
